# Mixture of Doxycycline, ML-7 and L-NAME Restores the Pro- and Antioxidant Balance during Myocardial Infarction—In Vivo Pig Model Study

**DOI:** 10.3390/biomedicines12040784

**Published:** 2024-04-02

**Authors:** Iwona Bil-Lula, Wiktor Kuliczkowski, Anna Krzywonos-Zawadzka, Piotr Frydrychowski, Dominika Stygar, Kornela Hałucha, Agnieszka Noszczyk-Nowak

**Affiliations:** 1 Department of Medical Laboratory Diagnostics, Division of Clinical Chemistry and Laboratory Haematology, Wrocław Medical University, 50-556 Wrocław, Poland; anna.krzywonos-zawadzka@umw.edu.pl (A.K.-Z.); kornela.halucha@student.umw.edu.pl (K.H.); 2 Institute of Heart Diseases, Wroclaw Medical University, 50-556 Wroclaw, Poland; wiktor.kuliczkowski@umw.edu.pl; 3 Department of Internal Medicine and Clinic of Diseases of Horses, Dogs and Cats, Faculty of Veterinary Medicine, Wrocław University of Environmental and Life Sciences, Grunwaldzki Square 47, 50-366 Wrocław, Poland; piotr.frydrychowski@upwr.edu.pl (P.F.); agnieszka.noszczyk-nowak@upwr.edu.pl (A.N.-N.); 4 Department of Physiology, Faculty of Medical Sciences in Zabrze, Medical University of Silesia, Jordana 19 Street, 41-808 Zabrze, Poland; dstygar@sum.edu.pl; 5 SLU University Animal Hospital, Swedish University of Agricultural Sciences, SE-750 07 Uppsala, Sweden

**Keywords:** ischemia-reperfusion injury, oxidative stress, MI pig model, pro- and antioxidant balance

## Abstract

The restoration of blood flow to the ischemic myocardium inflicts ischemia/reperfusion (I/R) heart injury (IRI). The main contributors to IRI are increased oxidative stress and subsequent excessive production of ROS, increased expression of NOS and peroxinitate, activation of MMPs, and enhanced posttranslational modifications of contractile proteins, which make them more susceptible to proteolytic degradation. Since the pathophysiology of IRI is a complex issue, and thus, various therapeutic strategies are required to prevent or reduce IRI and microvascular dysfunction, in the current study we proposed an innovative multi-drug therapy using low concentrations of drugs applied intracoronary to reach microvessels in order to stabilize the pro- and antioxidant balance during a MI in an in vivo pig model. The ability of a mixture of doxycycline (1 μM), ML-7 (0.5 μM), and L-NAME (2 μM) to modulate the pro- and antioxidative balance was tested in the left ventricle tissue and blood samples. Data showed that infusion of a MIX reduced the total oxidative status (TOS), oxidative stress index (OSI), and malondialdehyde (MDA). It also increased the total antioxidant capacity, confirming its antioxidative properties. MIX administration also reduced the activity of MMP-2 and MMP-9, and then decreased the release of MLC1 and BNP-26 into plasma. This study demonstrated that intracoronary administration of low concentrations of doxycycline in combination with ML-7 and L-NAME is incredibly efficient in regulating pro- and antioxidant balance during MI.

## 1. Introduction

Although substantial progress has been made in recent decades in reducing mortality and performing optimal revascularization in patients with myocardial infarction (MI), ischemic heart disease, including acute coronary syndrome (ACS), still remains the leading cause of mortality worldwide. Percutaneous coronary intervention (PCI) is the most frequently performed revascularization procedure worldwide [1]. However, restoration of blood flow to the ischemic myocardium inflicts additional ischemia/reperfusion (I/R) heart injury (IRI) [2]. In turn, IRI causes a variety of metabolic, morphological and contractile disorders, leading to irreversible microvascular damage or myocardial stunning [3]. This phenomenon may account for as much as 50% of the final MI size, a major determinant of the prognosis in patients with ST-elevation myocardial infarction (STEMI) [4,5]. Additionally, myocardial no-reflow can still occur and is associated with a worse in-hospital and long-term prognosis. The concept of no-reflow refers to a state of myocardial tissue hypoperfusion and microcirculatory dysfunction within a patent epicardial coronary artery [3]. Such patients with normal epicardial flow but impaired microvascular perfusion also have a high risk of developing congestive heart failure, arrhythmia, and death [6]. For this reason, one of the challenges nowadays is to better detect, prevent, and treat extended myocardial damage despite angiographically optimal revascularization.

Currently, the most important goal of pharmacological prevention and therapy of MI in clinical practice is to improve the oxygen supply/demand ratio for the heart. Nowadays, more than 90% of patients with suspicion of acute MI are given aspirin and/or β-blockers [7]. As there is no universally accepted pharmacological approach that gives satisfactory protection from, or treatment of, IRI, new directions in the pharmacological normalization of oxidative stress to reduce heart injury are still desirable.

The pathophysiology of IRI is a complex issue, and thus, it requires multisite actions to achieve the desired therapeutic effect. Oxidative stress leads to activation of high-output iNOS (inducible NOS) and consequently to overproduction of NO, but simultaneously to limited bioavailability of NO [8]. Physiological amounts of NO and its metabolites (nitrites) mediate cardioprotection [9], due to its antioxidant, vasodilator, anti-inflammatory, and anti-platelets effects [10]. However, oxidative stress during IRI triggers the increased production of very potent ONOO^-^ which promotes cell damage [11]. It was previously established that increased generation of ONOO^-^ during I/R leads to overactivation of matrix metalloproteinase-2 (MMP-2), and degradation of contractile proteins in consequence. Our colleagues previously discovered that massive oxidative stress induces phosphorylation and nitration/nitrosylation of myosin light chains (MLC1 and MLC2) [12,13,14,15], increasing their degradation by MMP-2 and contractile dysfunction. Additionally, we showed that inhibition of these modifications by ML-7 (an inhibitor of MLCK-myosin light chain kinase) or L-NAME (an inhibitor of NOS) and reduction of MMP-2 activity by doxycycline protected contractile proteins against degradation [14,16].

Given that both MMP-2 activity and MLC modifications have physiological roles, a full pharmacological blockade would result in a multitude of physiological side effects and cardiac cytotoxicity that may be as detrimental as the IRI itself. Thus, in the previous study, we proposed the combination of lower concentrations of therapeutic agents, which may afford similar, if not improved, protection while limiting adverse side effects [17,18,19,20]. It has been shown that these agents presented different modes of action and can synergistically or additively protect the heart from IRI.

Since the pathophysiology of IRI is a complex issue, and thus, it requires multisite actions to achieve the desired therapeutic effect, here, the aim of the current study was to test the multi-drug therapy (doxycycline + ML-7 + L-NAME) using the low concentrations of drugs applied indirectly to heart microvessels in order to stabilize the pro- and antioxidant balance during I/R in an in vivo pig heart model.

## 2. Materials and Methods

### 2.1. Study Group

This randomized trial was performed at Wroclaw Medical University in cooperation with Wroclaw University of Environmental and Life Sciences, Wrocław, Poland. This investigation conformed to the National Institute of Health’s guidelines for the care and use of the laboratory animals. The study was approved by the Local Ethics Committee for Experiments on Animals, no. 081/2019 (approved on 11 December 2019).

Aged 16–20 weeks female pigs (Sus scrofa domestica) of the Polska Biała Zwisłoucha breed (35–45 kg weight) n = 12, purchased from the Experimental Station of the National Research Institute of Animal Production in Żerniki Wielkie (Poland), participated in the study. As in our previous studies using a porcine model, the use of female pigs was dictated by their greater resilience to stress resulting from new housing conditions, as well as daily handling and grooming. They are also more resistant to perioperative stress and less aggressive towards other animals and staff. Moreover, the menstrual cycle of female animals does not affect the results of the experiments, as the females at the time of the experiment had not yet reached reproductive maturity.

During the study period, the animals were housed and maintained in controlled conditions of prosperity to eliminate stress (2 weeks of acclimatization), their diet conformed to nutritional standards, and they had water ad libitum. The animals were habituated to grooming activities before the study started.

The participants were not considered for enrollment if they presented atrial fibrillation or sinus tachycardia of more than 200 bmp. Furthermore, subjects were excluded if they met one of the following exclusion criteria: finding a congenital defect in echocardiography, shortening fraction (SF) below 25% or ejection fraction (EF) below 40%, significant mitral or tricuspid regurgitation, WBC up to 25 G/L, or renal or hepatic failure.

The information provided in the paper complies with the ARRIVE guidelines.

### 2.2. Experimental Treatment Protocol and Sample Size

Consequently, randomization to either the control (MI) or tested mixture of drugs (MI-MIX group) was performed prior to angiography in all subjects without pre-angiographic exclusion criteria using a 1:1 computer-generated sequence. The following groups were tested:

(1) The myocardial infarction group with an intracoronary infusion of DOXY (1.0 µM) + ML-7 (0.5 µM) + L-NAME (2 µM) diluted in saline by an infusion catheter distal to the angioplasty site constituted the MI-MIX (n = 6).
(2) The myocardial infarction group with an intracoronary infusion of the same volume of 0.9% NaCl constituted the sham-MI control group (n = 6).

In terms of statistical verification of the results (Student’s *t*-test, two-way ANOVA with contrast analysis, analysis of variance for repeated measures, Mauchly’s test), the number of animals planned in the group (n = 6) and the number of groups (n = 2) were limited to the level necessary to achieve the project goals and are the smallest possible. These were determined based on test power analysis, assuming *p* ≤ 0.05 and test power = 0.8. Group sizes were determined using the calculator at http://biomath.info/power/ttest.htm (accessed on 1 September 2019), taking into account the test’s power analysis. Following the replacement, reduction, and refinement concept, the project’s animal count was restricted to that which was required to meet its objectives and address the study question.

### 2.3. Premedication and Monitoring

The pigs underwent dietary restriction from solids for 12 h to eliminate gastric contents before the intervention and were maintained with ad libitum water consumption. The pigs eligible for catheterization were pretreated with Vetaketam (10 mg/kg b.m. i.m., Vet-Agro Sp. z.o.o., Lublin, Poland), Midazolam (0.3 mg/kg b.m. i.m., Polfa Warszawa S.A., Warszawa, Poland), and Sedator (0.03 mg/kg b.m., Eurovet Animal Health BV, Bladel, The Netherlands). The general anesthesia was induced with an intravenous (marginal vein of the ear catheterization) bolus 2 mg/kg b.m. of propofol (Provive, Claris Lifesciences UK Ltd., Cheshire, UK).

Once sufficiently sedated, the pigs were subjected to tracheal intubation (size 8, Tracheal Tube type Murphy, SUMI, Sulejówek, Poland) and subsequently ventilated with 100% oxygen and a closed gas system with a carbon dioxide absorber. Ventilation parameters were as follows: the oxygen flow was 2 L/min, the tidal volume was 10 mL/kg b.m., and the respiratory rate was 12 L/min. The end-tidal CO_2_ concentration was maintained between 35 and 45 mmHg.

Anesthesia was maintained with isoflurane (1.5–2.5 vol%, Forane, Abbott Laboratories, Warszawa, Poland) by inhalation (Vapor 2000, Dräger Medical AG & Co. KGaA, Lübeck, Germany). An intravenous bolus (10 µg/kg b.m.) of fentanyl (Fentanyl WZF 50 µg/mL, Polfa Warszawa S.A., Warszawa, Poland) and then the constant infusion of 10 µg/kg b.m. /h rate provided analgesia. If any hypotension events occurred, 10 mL/kg b.m. boluses of lactated Ringer’s solution (Solutio Ringeri Lactate Fresenius, Fresenius Kabi Polska Sp. z o.o., Warszawa, Poland) and 3–5 mL/kg b.m. boluses of hydroxyethyl starch 130/0.4 (HES; Voluven, Fresenius Kabi Deutschland GmBH, Bad Homburg vor der Höhe, Germany) were administered to stabilize the pressure.

Body temperature was measured to maintain 36 °C. Continuous 12-lead ECG (BTL-08 MT Plus ECG, BTL Industries Ltd., Hertfordshire, UK) monitoring during the procedure allowed us to control the incidence and type of ventricular arrhythmias. Respiratory and hemodynamic parameters were recorded, inter alia: breath, saturation, heart rate, and ECG.

### 2.4. Angiography, STEMI Induction, and Reperfusion

**Figure 1:**
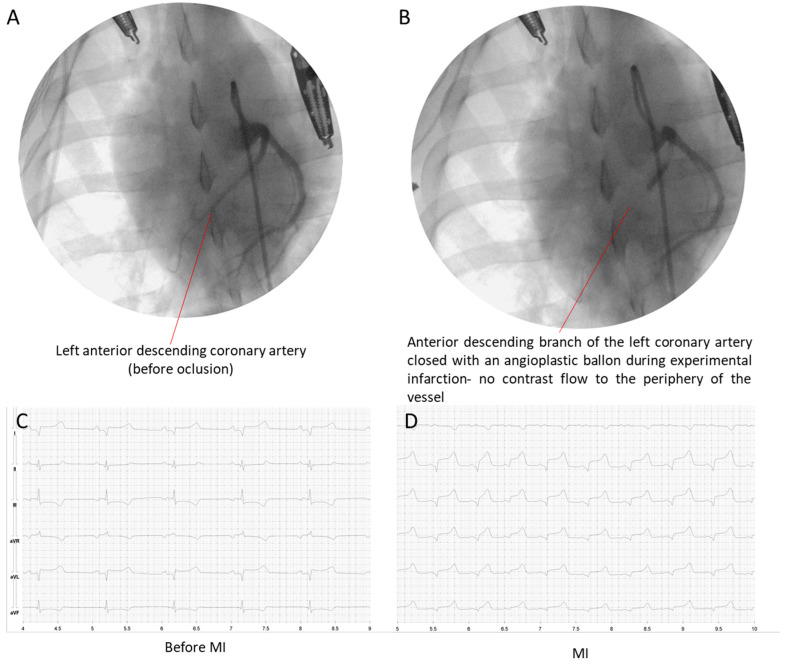
Angiographic image of the left anterior descending artery before ischemia (**A**) and at the time of occlusion (**B**). Representative ECG recording confirming normal heart’s electrical activity before (**C**) and during (**D**) myocardial (MI) infarction.

Coronary angiography was performed prior to angioplasty during the same anesthesia. Vascular access for the procedure was by default right common femoral artery punctured under ultrasound guidance (F37, Hitachi Aloka Medical Ltd., Shanghai, China). Vascular assess was obtained with 6F vascular sheath (Balton, Warszawa, Poland) and a puncture needle (21G, Balton, Warszawa, Poland). Endovascular, fluoroscopy-guided (Symbol, General Medical Merate SpA, Seriate, Italy), coronary procedures (BMW, 3 m, 0.014″, Abbott, Green Oaks, IL, USA) were performed to occlude the left anterior descending artery (LAD) (30 min) by using a coronary angioplasty balloon. Briefly, by using a portable radiologic source for fluoroscopy guidance, the left main coronary artery was intubated with a 6-French guiding Judkins left 3.5 catheter (Launcher, JL 3.5 curvatures, 6F diameter, Medtronic, Minneapolis, MN, USA), then the 6000 UI heparin bolus (Heparinum WZF, Polfa Warszawa S.A., Warszawa, Poland) was injected, and baseline coronary angiography was performed. A standard angioplasty guide wire was introduced to the anterior descending artery (Figure 1A). An angioplasty balloon (3.0 × 10 mm) (Sprinter, OTW model, Medtronic, Minneapolis, MN, USA) was placed in the proximal segment of LAD, inflated to a pressure of 6 atm for 30 min to obtain complete LAD occlusion (Figure 1B). STEMI was defined as significant ST-segment elevation in at least two contiguous leads on the 12-lead ECG (Figure 1C,D).

After occlusion, the occlusive balloon catheter was deflated to achieve reperfusion. Ten min before the reperfusion study, a mixture of drugs (DOXY (1.0 µM) + ML-7 (0.5 µM) + L-NAME (2 µM) (MI + MIX group) or 0.9% NaCl (MI group)) was injected intracoronary, to omit the typical drug distribution pathways in the body and to better achieve cardiac tissue. After the whole procedure, all catheters, balloons and guide wires, together with both femoral vascular sheaths, were withdrawn, and access sites were closed with a compression dressing, anesthesia was suspended, and the pigs were weaned from mechanical ventilation and then transferred to the post-surgery recovery suite. All participants were treated with amiodarone (5 mg/kg/24 h) before PCI. If VF occurred, defibrillation was applied immediately.

### 2.5. Postoperative Treatment and Maintaining

**Figure 2:**
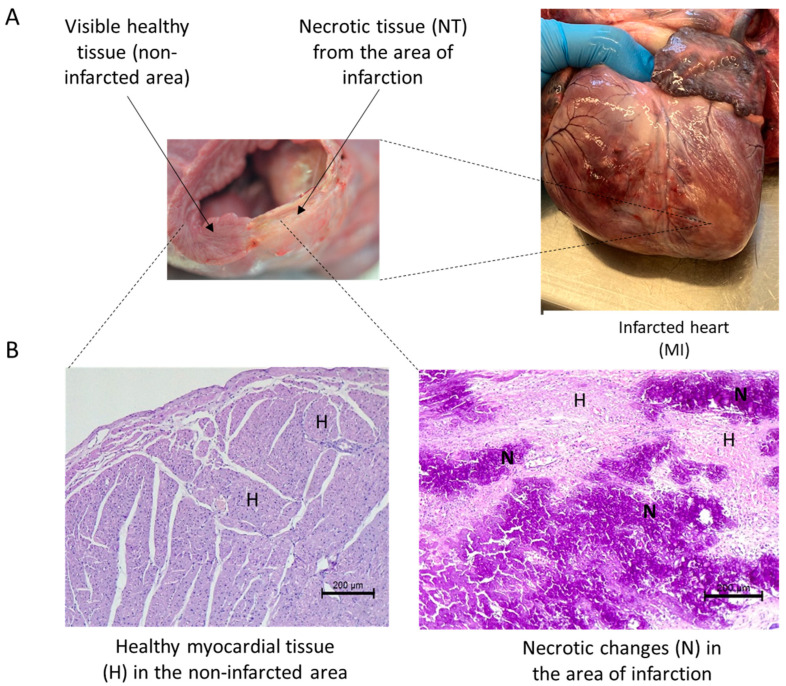
Representative images of the heart subjected to myocardial infarction show a macroscopic evaluation of necrotic tissue in the MI area and healthy tissue (**A**). Representative microscopic images of HE stained tissue from infarcted and non-infarcted areas of the heart (**B**), magnification factor 200 µm. H—healthy myocardial tissue; N—necrotic changes in the area of infarction.

An analgesic treatment was provided postoperatively, by intramuscular injections of buprenorphine (0.01 mg/kg b.m., every 8 h) for 3 days (Bupaq Multidose, Richter Pharma AG, Wels, Austria), metamizole 50 mg/kg b.m., every 24 h for 3 days (Pyralgivet, Vet-Agro Sp. z.o.o., Lublin, Poland), and meloxicam (0.4 mg/kg b.m., every 24 h) for 2 days (Metacam, Boehringer Ingelheim Vetmedica GmbH, Rohrdorf, Germany). Until the fourteenth day after the procedure, the pigs were housed and maintained as before the experiment. Then, the animals were euthanized after 14 days of follow-up, with pentobarbital (100 mg/kg, i.v.; Morbital, Biowet Puławy Sp z o.o., Puławy, Poland). Heart tissue from the infarcted (necrotic tissue from the area of infarction) and non-infarcted (visible healthy tissue) areas of the left ventricular myocardium was collected from all hearts in both groups (n = 12 in total, n = 6 per group) for further analyses during the autopsy (Figure 2A). In addition to the visual (macroscopic) evaluation of the tissues, the necrotic and healthy tissue areas were confirmed microscopically. HE staining was used to assess the extent of the scar (Figure 2B).

### 2.6. ECG

The ECG examination was performed in two positions: the right lateral position and next on the back, as previously described [21]. Briefly, for assurance of a stable and repeatable position of the body on the back, a radiological positioner was used. Recordings were collected and analyzed using a 12-lead BTL-08 MT Plus resting ECG system with BTL-08 Win 6.14 software support (BTL Industries Ltd., Hertfordshire, UK). Due to the lack of literature data on the electrode placement system for swine, precordial electrodes were placed as recommended by Santilli et al. [22] for dogs with a similar chest structure. Limb electrodes were placed in a standard system [23].

### 2.7. Collection and Analysis of Blood Samples

Blood samples were collected during catheterization of right femoral artery shortly before coronary artery occlusion (T0), during the reperfusion (TR) 15 min after deflating the angioplasty balloon and 25 min after MIX administration, and at the end of 14 days follow-up (TE) (external jugular vein puncture) to test oxidative stress and antioxidative markers in the blood.

### 2.8. Heart Tissue Homogenization

A total of 100 mg of cardiac tissue from the left ventricle was homogenized in homogenizing buffer with protease inhibitors (1:10 *w*/*v*) with a glass homogenizer (Potter-Elvehjem PTFE, Sigma-Aldrich, Darmstadt, Germany) and then sonicated (Virsonic 100, VirTis, Gardiner, NY, USA). The lysate was centrifuged for 10 min, at 4000 rpm, at 4 °C, and treated as one independent sample. The tissue samples were then frozen and stored at −80 °C until the analysis.

### 2.9. ELISA Tests

Commercial ELISA tests were used to quantitatively evaluate the porcine MMP-2 (ABIN6015984, Antibodies-Online, Pottstown, PA, USA), MMP-9 (EKC38274, Biomatik, Cambridge, ON, Canada), MLC1(ABIN6231417, Antibodies-Online, Pottstown, PA, USA), and BNP (S-1190, Penisula Laboratories International, Inc., San Carlos, CA, USA) production in serum.

### 2.10. Total Antioxidant Capacity

Randox TAS assay kit (Randox Co., Antrim, UK) was used to assess total antioxidant capacity (TAC) in heart homogenates. Briefly, a translucent reduced ABTS molecule (2,2′-azino-bis(3-ethylbenzothiazoline-6-sulfonate) was oxidized to blue–green ABTS+. Mixing ABTS+ with any substance that can be oxidized, reversed ABTS+ to its original translucent form. Results were expressed in mmol/L [24].

### 2.11. Total Oxidative Status

Total oxidative status (TOS) was measured by the method that utilizes the oxidation of Fe^2+^ to Fe^3+^ in an acidic medium, as previously described [25]. The produced Fe^3+^ ions react with xylene orange and form a colorful blue–purple complex, that shows maximum absorbance at 560 nm. The TOS level was read from the calibration curve with H_2_O_2_ as the standard, and the results were expressed in μmol/L.

### 2.12. Oxidative Stress Index

The oxidative stress index (OSI) was expressed as the combined ratio of the total oxidant status (TOS) to the total antioxidant capacity (TAC) in arbitrary units to the following formula: OSI = [(TOS, mmol/L)/(TAS, mmol/L)] [26].

### 2.13. Malondialdehyde Concentration

The method with the thiobarbituric acid described by Ohkawa et al. was used to determine malondialdehyde (MDA) concentration in heart tissue [27]. MDA concentration was detected spectrophotometrically (excitation 515 nm, emission 552 nm). The standard curve was prepared using 1,1,3,3-tetraethoxypropane and expressed in μmol/L.

### 2.14. Glutathione Peroxidase Activity

Kinetic method developed by Mannervik was used to assess glutathione peroxidase (GPx) activity (EC 1.11.1.9). The method was based on spectrophotometric measurement of NADPH concentration decrease at 340 nm for 10 min. GPx activity was expressed in IU/mg protein [28].

### 2.15. Glutathione Reductase Activity

Carlberg and Mannervik’s kinetic method for glutathione reductase (GR) (EC 1.8.1.7) activity measurement was used to assess the decrease in NADPH concentration in the samples at 340 nm for 10 min. GR activity was expressed in IU/mg protein [29].

### 2.16. Glutathione S-Transferase Activity

The kinetic method described by Habig and Jakoby [9] with 1-chloro-2,3-dinitro-benzene as a reaction substrate was used to evaluate glutathione S-transferase (GST) activity (EC 2.5.1.18). GST activity was expressed in IU/mg protein.

### 2.17. Lipofuscin Concentration

Lipofuscin (LF) as a marker of mitochondrial ROS formation was evaluated in the serum as follows: the serum sample was mixed with ethanol-ether (3:1, *v*/*v*), shaken out, and centrifuged. Then, the fluorescence intensity of the supernatant was determined at 345 nm (absorbance) and 430 nm (emission). LPS concentration was expressed as relative lipid extract fluorescence (RF), since 100 RF corresponds to the fluorescence of 1 μg/mL quinidine sulfate in 0.1 N sulfuric acid. The method is described in details by Tsuchida et al. [30].

### 2.18. Statistical Analysis

Statistical analysis was performed using Statistica, version 13 (TIBCO Software Inc., Palo Alto, CA, USA). Shapiro–Wilk test was used to check the distribution of the data. The mean ± standard deviation (SD) was calculated for normally distributed data, and the median with an upper and lower quartile was analyzed when a skewed distribution was observed. The data with a skewed distribution were log-transformed before further analyzes. A two-way ANOVA analysis with contrast analysis was used for concentrations comparison in the heart tissues samples. An analysis of variance for repeated measures was used to compare the concentrations in the serum samples, and the Mauchly’s test was used to assess the sphericity assumption. Pearson correlation was used to correlate serum and tissue parameters. Statistical significance was set at *p* < 0.05.

## 3. Results

### 3.1. Total Oxidative Status in Heart Tissue

**Figure 3:**
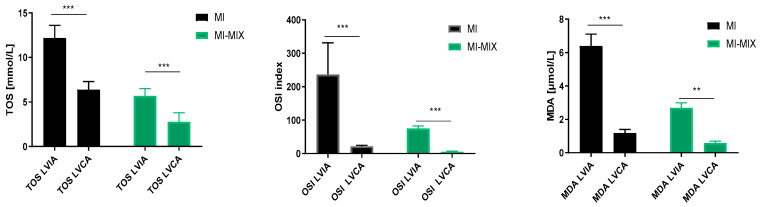
Changes in oxidative status in left ventricle heart tissue from infarct area and non-infarct area, independently of drug administration. Notes: TOS—total oxidative status; OSI—oxidative stress index; MDA—malondialdehyde; LVIA—left ventricle heart tissue from infarct area; LVCA—left ventricle heart tissue from non-infarct area (control); MI—myocardial infarction; MI-MIX—myocardial infarction with administration of MIX; MIX—mixture of doxycycline (1 μM), ML-7 (0.5 μM) and L-NAME (2 μM); ***—*p* < 0.001, **—*p* < 0.01.

The total oxidant status (TOS), oxidative stress index (OSI), and malondialdehyde (MDA) were evaluated in the left ventricle heart tissue from the infarct area and compared to the left ventricle heart tissue from the non-infarct area. Infarction in the myocardium induced a significant increase in TOS (*p* < 0.001), OSI (*p* < 0.001), and MDA (*p* < 0.001), independently of drug administration (Table 1 and Figure 3).

### 3.2. Total Antioxidative Status in Heart Tissue

**Figure 4:**
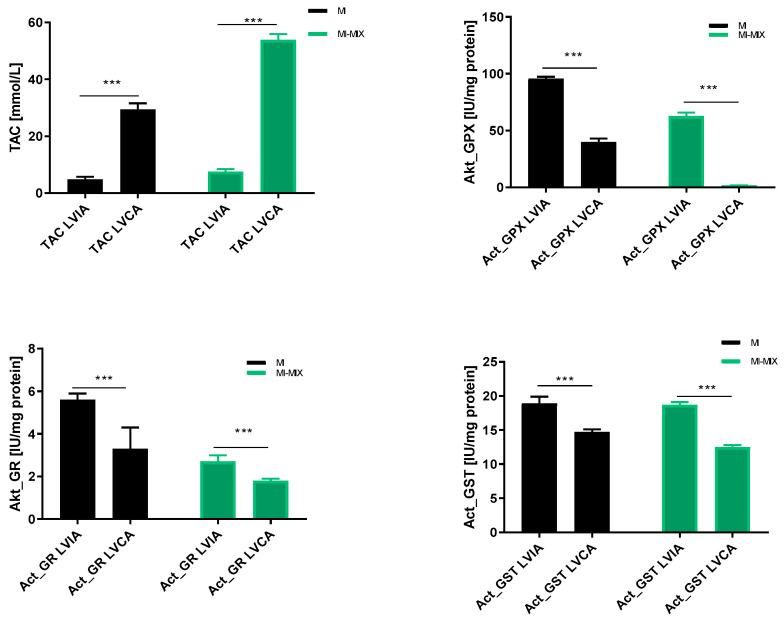
Changes in antioxidative status in left ventricle heart tissue from an infarct area and a non-infarct area independently of drug administration. Notes: TAC—total antioxidant capacity; act_GPx—activity of glutathione peroxidase; act_GR—activity of glutathione reductase; act_GST—activity of glutathione S-transferase; LVIA—left ventricle heart tissue from infarct area; LVCA—left ventricle heart tissue from non-infarct area (control); MI—myocardial infarction; MI-MIX—myocardial infarction with administration of MIX; MIX—mixture of doxycycline (1 μM), ML-7 (0.5 μM) and L-NAME (2 μM); ***—*p* < 0.001.

Total antioxidant capacity (TAC), glutathione peroxidase (aktGPX), glutathione reductase (aktGR), and glutathione S-transferase (GST) were evaluated in the left ventricle heart tissue from the infarct area and compared to the left ventricle heart tissue from the non-infarct area. Data showed that the TAC was significantly decreased in the infarct area compared to the non-infarcted zone (*p* < 0.001), but the activity of the GPX, GR, and GST increased during myocardial infarction, independently of the drug supply. The results have been presented in Table 2 and Figure 4.

**Figure 5:**
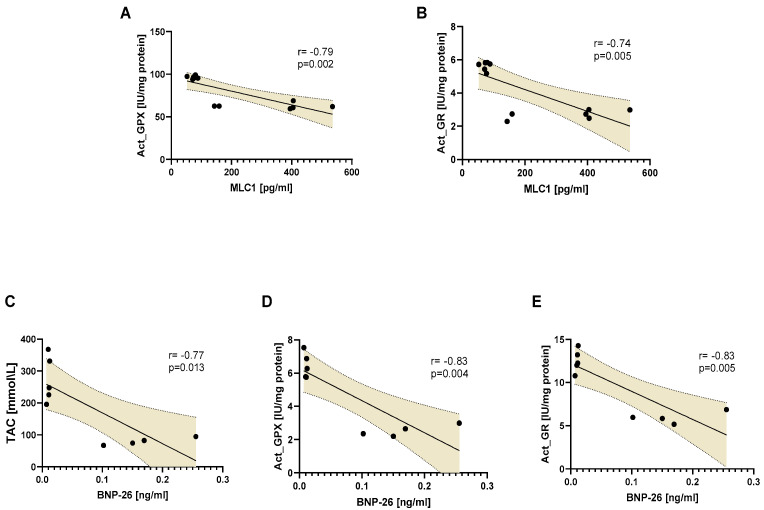
The correlation between tissue antioxidative markers and the release of MLC1 or BNP-26 into the plasma: act_GPX vs. MLC1 (**A**), act_GR vs. MLC1 (**B**), TAC vs. BNP-26 (**C**), act_GPX vs. BNP-26 (**D**), and act_GR vs. BNP-26 (**E**). Notes: TAC—total antioxidant capacity; act_GPx—activity of glutathione peroxidase; act_GR—activity of glutathione reductase; act_GST—activity of glutathione S-transferase; MLC1—myosin light chains type 1; BNP-26—B-type natriuretic protein.

Increased TAC, act_GPX, act_GR, as markers of antioxidative balance, were correlated with decreased concentrations of MLC1 or BNP-26 in porcine plasma (Figure 5).

### 3.3. An Influence of MIX Treatment on Oxidative and Antioxidative Status in Heart Tissue

**Figure 6:**
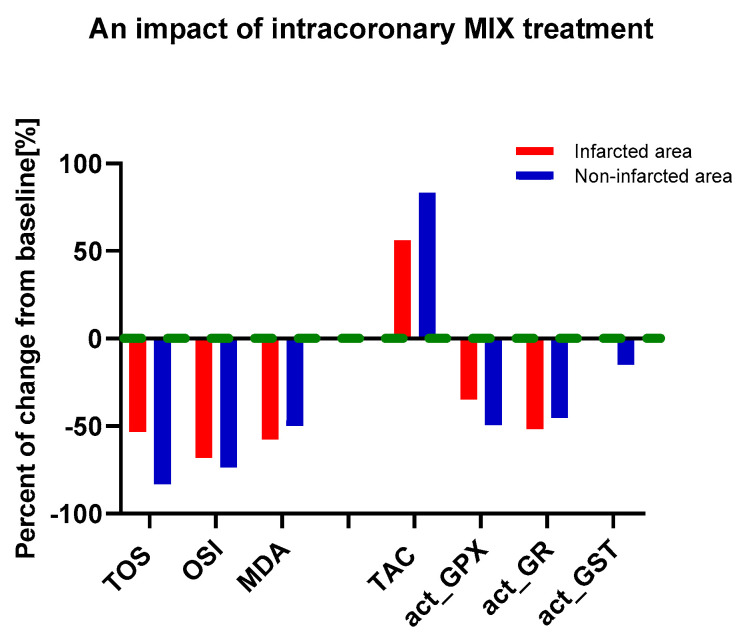
Percentage impact of intracoronary MIX treatment on changes in pro- and antioxidant status. Notes: TAC—total antioxidant capacity; TOS—total oxidative status; OSI—oxidative stress index; MDA—malondialdehyde; act_GPX—activity of glutathione peroxidase; act_GR—activity of glutathione reductase; act_GST—activity of glutathione S-transferase; MIX—mixture of doxycycline (1 μM), ML-7 (0.5 μM); baseline (green) refers to the tissue not treated with MIX.

TOS, OSI, MDA, as well as TAC, act_GPX, act_GR and act_GST were evaluated in the heart tissue subjected to a MIX treatment as compared to non-treated tissue. Data showed that an intracoronary infusion of a MIX significantly reduced the TOS (53.3%), OSI (68.1%), and MDA (57.8%) in the area of the MI and consequently the TOS (83.3%), OSI (73.7%) and MDA (50%) in the control tissue. Moreover, intracoronary infusion of a MIX significantly increased the TAC both in the area of the MI (56.3%) and in the control tissue (83.3%). Administration of a MIX also significantly decreased act_GPX (35% and 49.4%) and act_GR (51.8% and 45.5%) in the area of the infarcted and control tissue, respectively. The mixture had the least effect on the GST, since data showed no effect in the infarcted area and weak lowering in the control tissue (15%) (Table 3, Figure 6).

Contrast analysis confirmed that myocardial infarction decreased the TAC and increased the TOS, OSI, MDA, as well as the GPX, GR and GST activity in comparison to control. However, intracoronary administration of the MIX reversed this effect. Moreover, independently of the MI, administration of the MIX led to increased TAC and decreased TOS, OSI, MDA, as well as GPX, GR and GST activity (Table 4).

### 3.4. Oxidative and Antioxidative Parameters in Serum

**Figure 7:**
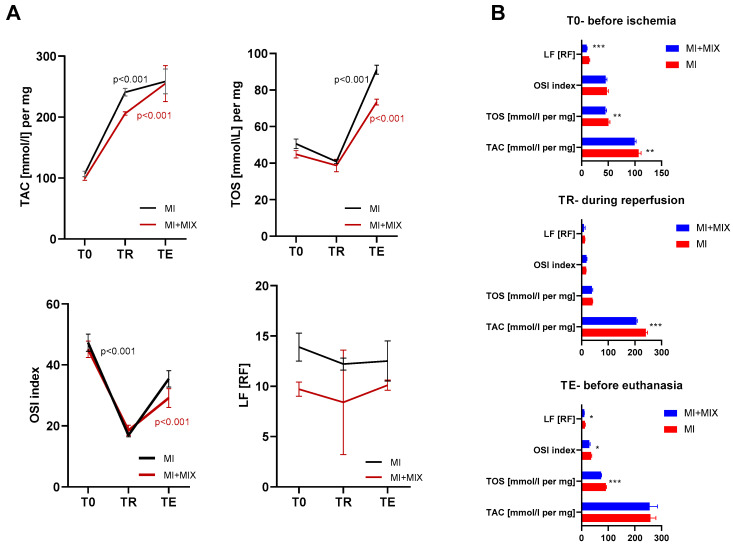
Serum concentrations (**A**) and MIX treatment effect (**B**) on serum concentrations of pro- and antioxidant markers at different time points. Notes: TAC—total antioxidant capacity; TOS—total oxidative status; OSI—oxidative stress index; LF—lipofuscin; MI—myocardial infarction; MIX—mixture of doxycycline, ML-7 and L-NAME; T0—before ischemia; TR—during reperfusion; TE—before euthanasia; ***—*p* < 0.001; **—*p* < 0.01; *—*p* < 0.05.

Analysis of antioxidative (TAC) and prooxidative stress markers (TOS, OSI, LF) in serum showed a significant increase of the TAC during reperfusion as well as further increase up to euthanasia, both in the group subjected only to the MI and those subjected to the MI and MIX treatment. Inversely, the TOS as well as the OSI index decreased in blood during reperfusion (TR), then rose again before euthanasia (TE). In turn, the LF did not reveal significant changes during the time (Figure 7A). Analysis of the influence of the MIX treatment on the parameters of the pro- and antioxidative status showed that intracoronary administration of an inhibitory mixture led to decreased TAC as well as TOS and LF before ischemia. Moreover, the MIX decreased the TAC during reperfusion, as compared to the higher levels during reperfusion without drugs. Follow-up until 14 days after the MI showed decreased TOS, OSI, and LF during the MIX administration (Figure 7B).

### 3.5. An Influence of Doxy, ML-7, and L-NAME Mixture on Proteins Release into Blood

**Figure 8:**
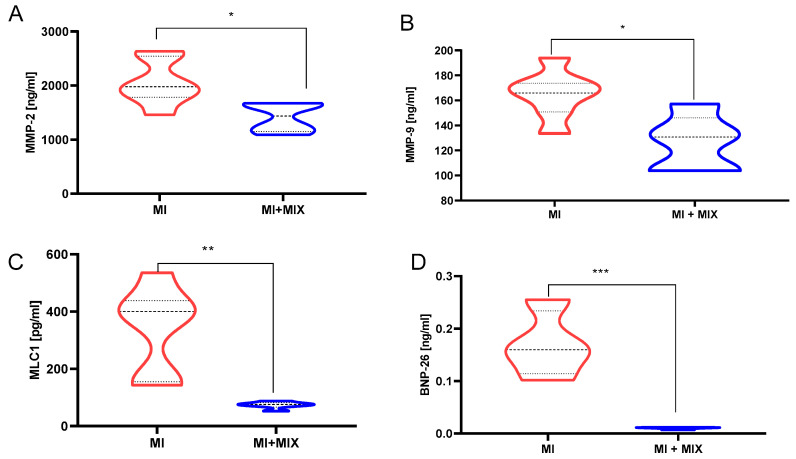
Analysis of MMP-2 (**A**), MMP-9 (**B**), MLC1 (**C**), and BNP-26 (**D**) in porcine plasma at reperfusion. Notes: MMP-2/MMP-9—matrix metalloproteinases 2 and 9; MI—myocardial infarction; MI-MIX—myocardial infarction with administration of MIX; MIX—mixture of doxycycline (1 μM), ML-7 (0.5 μM), and L-NAME (2 μM); ***—*p* < 0.001; **—*p* < 0.01; *—*p* < 0.05.

Analysis of blood samples collected during reperfusion (TR) showed an abundant synthesis and release of MMP-2 (2048.0 ± 419.0 ng/mL) and MMP-9 (165.1 ± 19.07 mg/mL) in the MI group, followed by increased degradation and extracellular release of cardiac proteins such as MLC1 (340.7 ± 155.7) and BNP-26 (0.16 ± 0.064 mg/mL) (Figure 8). Intracoronary administration of the Doxy, ML-7, and L-NAME mixture during ischemia led to a decrease in MMP-2 (*p* = 0.0134), MMP-9 (*p* = 0.017), MLC1 (*p* = 0.003), and BNP-26 (*p* = 0.002) concentrations in the blood as compared to the MI.

## 4. Discussion

Translational medicine demands selected models depending on the particularities of the human diseases to be investigated, reproducing as closely as possible the evolution, clinical symptoms, and molecular pathways, cells, or tissues involved in the dysfunction. Therefore, the pig model offers an alternative because of the pig’s anatomical and physiological similarities to humans and the availability of genomic, transcriptomic, and progressively more proteomic tools for analysis of this species [7,31]. Moreover, the pig model allows for the use of instrumentation exactly as in humans, reflecting the conditions of possible surgical therapy in people with MI. For this reason, a pig model of a MI was included in this study to estimate the influence of a multi-drug therapy using low concentrations of drugs on pro- and antioxidative balance in heart tissue during myocardial infarction.

Coronary microvessels play an essential role in supplying oxygen and nutrients to the the myocardium by regulating coronary flow conductance and substance transport. Although nowadays, interventional cardiologists can treat acute epicardial artery occlusion effectively, myocardial damage can remain high, as a result of microcirculation impairment [32]. For this reason, the tested mixture was directly applied intracoronary to reach microvessels and balance the pro- and antioxidant status. Intracoronary administration of the tested mixture also allowed for direct application of drug doses established as cardioprotective in a MI within rat hearts tested ex vivo.

The main contributors to IRI are increased oxidative stress [13] and subsequent excessive production of ROS [33], increased expression of NOS [34], activation of MMPs [35], and enhanced posttranslational modifications of contractile proteins, which make them more susceptible to proteolytic degradation [36]. Oxidative stress leads to activation of high-output iNOS and consequently to overproduction of NO, but simultaneously to limited bioavailability of NO [8]. Physiological amounts of NO and its metabolites (nitrites) mediate cardioprotection [9] due to their antioxidant, vasodilator, anti-inflammatory, and anti-platelets effects [10]. However, oxidative stress during IRI triggers the increased production of very potent ONOO^-^ which promotes cell damage [37]. Peroxynitrite also causes nitration/nitrosylation of myocardial proteins [38] and activates MMP-2 [17], leading to enhanced degradation of proteins and contractile dysfunction [35]. Perkins et al. have shown that attenuating uncoupled eNOS during reperfusion reduced oxidative stress and led to the restoration of cardiac function [39]. In our previous studies in an ex vivo rat heart global ischemia model, we showed that simultaneous inhibition of MMP-2 (by doxycycline), myosin light-chain kinase- MLCK (by ML-7) and iNOS/eNOS (by L-NAME) served as cardioprotection [17,18]. We showed the synergistic effect of subthreshold concentrations of tested drugs on the contractility of hearts subjected to I/R. We also proved that administration of Doxy (1 μM), ML-7 (0.5, μM), and L-NAME (2 μM) into rat hearts subjected to global ischemia followed by aerobic reperfusion resulted in the full recovery of cardiac mechanical function, as a result of significantly increased coronary flow as well as reduced heart injury [18]. Additionally, we proved that the proposed mixture normalized the pathway of iNOS/eNOS/phospho-eNOS/ADMA/NO during I/R, thus reducing oxidative stress [17]. Hence, since the pathophysiology of IRI is a complex issue, and thus, various therapeutic strategies are required to prevent or reduce IRI and microvascular dysfunction, in the current study we proposed an innovative multi-drug therapy using low concentrations of drugs applied intracoronary to reach microvessels in order to stabilize the pro- and antioxidant balance during a MI.

In the past two decades, a large amount of studies have pointed to reactive oxygen species (ROS) in the development of cardiovascular diseases [40,41,42,43]. The main and most biologically active ROS are superoxide (O2•−) and hydrogen peroxide (H_2_O_2_), produced by numerous oxidases [44,45]. The third one is NO•, produced by both eNOS and iNOS [10]. Their interactions induce the production of further reactive oxygen species such as peroxynitrite (ONOO^-^), which injures vascular and myocardial cells [46]. Whereas low levels of ROS, as a powerful signaling molecule, are necessary for maintaining normal vascular function, excess production of ROS or impaired ROS removal exacerbates oxidative stress and regulates the cellular mechanisms of response to injury [33,47,48].

In this study, we confirmed that myocardial infarction, generated by the occlusion of the left anterior descending artery, induced oxidative stress, expressed in the form of increased total oxidative status, oxidative stress index, and malondialdehyde concentration in the area of infarcted tissue, as compared to the non-infarcted area, in hearts treated and not treated with the mixture of drugs. However, intracoronary infusion of a MIX led to a reduction of the TOS, OSI and MDA, suggesting its antioxidative properties. The mixture of drugs also presented antioxidant properties in the area of non-infarcted heart tissue (LVCA), confirming its antioxidative potential independently of the factors inducing the generation of free radicals.

Since ROS can be endogenously formed between dioxygen and free electrons traveling through the respiratory pathway in the mitochondria, organisms need mechanisms to protect themselves from oxidative damage, so they have developed efficient protective networks based on a wide variety of antioxidants [49]. During the stress condition in the organism, there are at least several mechanisms counteracting the negative impact of ROS, for example: superoxide dismutase (SOD) [44,50], catalase [51], gluthation peroxidase (GPX) [52], thioredoxins [53,54], and peroxiredoxins [55,56]. Here, we reported significantly reduced total antioxidant capacity and increased activity of glutathione peroxidase, glutathione reductase and glutathione S-transferase in the area of the myocardial infarction, regardless of the drugs used. All of the above confirms an imbalance between pro- and antioxidants in the condition of myocardial infarction. Glutathione (GSH) is one of the most important cellular antioxidant systems. In its reduced form, it is capable of scavenging reactive oxygen and nitrogen species, thereby contributing to the control of redox homoeostasis. Glutathione peroxidases are a family of at least 8 isoforms of oxidoreductases that reduces H_2_O_2_ by the reduced form of glutathione [42,57]. Similarly, glutathione reductase (GR) regenerates intracellular GSH by reducing oxidized glutathione (GSSG) in the presence of NADPH and flavine adenine dinucleotide (FAD) [49]. On the other hand, glutathione S-transferases (GST) catalyzes the conjugation of the reduced form of glutathione (GSH) to endogenous and xenobiotic substrates for the purpose of detoxification [49,58]. Our results are compatible with other works, confirming dysregulation of pro- and antioxidants homeostasis during ischemia and reperfusion. Hill and Signal showed increased GPX at one week after a MI [59], and Zuzak et al. revealed increased serum GR in comparison to control patients. It was also confirmed that GPx-1 which is present in the mitochondria and the cytosol, is a critical enzyme in the protection of vessels from oxidative stress and atherogenesis [60]. For this reason, myocardial infarction in the tested pig model induced increased GPX and GR activity, which are essential for scavenging hydrogen peroxide and protecting cardiovascular cells. Our research also showed increased activity of GST during a MI. Taking into account that the absence of the functional GST enzyme [30] is associated with greater vulnerability to oxidative stress [61] and exacerbates ischemia–reperfusion injury [62], increased activity of GST during a MI may be involved in the reduction of oxidative stress and the reduction of ischemia-reperfusion injury. Additionally, intracoronary infusion of the inhibitory mixture led to a reverse of the changes occurring during infarction. We observed increased total antioxidant capacity and reduced activity of glutathione peroxidase and reductase in the infarcted area of the left ventricle heart tissue. Moreover, the same effect was observed in non-infarcted areas subjected to a MIX treatment. The treatment did not affect the reduced activity of GST, confirming no association with this route of detoxification.

The greatest impact of the tested mixture was observed on the pro-oxidation balance, since the intracoronary administration of Doxy, ML-7 and L-NAME reduced oxidative stress markers by 50.0–83.3%, regardless of the MI. It affects the total oxidative capacity and antioxidant enzymes with a similar effect, from 45.5–83.3%. The mixture had the weakest effect on GST activity (15.0%), and finally had no effect on GST in the infarcted tissue.

The concept of cardiac reperfusion injury is recognized as a clinical phenomenon that may result in microvascular damage or myocardial stunning. The precise contribution of different cellular and enzymatic sources of the ROS involved in cardiovascular pathologies remains unexplained. However, it was confirmed that an increased degradation of heart contractile proteins during I/R is caused by an increased proteolytic activity of intracellular enzymes. There are at least two proteins that have attracted considerable interest. One is matrix metalloproteinase-2 (MMP-2), whose increased activity was observed in hearts subjected to oxidative stress [12,13,35]. It is activated by ONOO^-^ [63,64,65,66,67] and degrades contractile proteins [14,68,69]. The second protein is myosin light chain (MLC), which is modified during acute ischemia and degraded by MMP-2 [16,36,70]. Interestingly, in 2018 Gerlach et al. showed for the first time that MMP-2 is also responsible for increased ROS formation and activation of signaling mechanisms impairing redox balance [55]. They also proved that the mechanism of increased ROS generation came from the fact that MMP-2 transactivates the heparin-binding epidermal growth factor (HB-EGF), leading to EGF receptor (EGFR) activation. For this reason, the use of MMP inhibitors such as doxycycline in the tested mixture might induce an antioxidative effect.

Given that both MMP-2, MLCK, and NOS activities, as well as MLCs modifications, have various physiological roles, full pharmacological blockade would result in a multitude of physiological side effects that may be as detrimental as the I/R injury itself. Thus, instead of using one drug at a therapeutic and relatively high concentration, we propose a combination of lower concentration therapeutic agents such as DOXY (1.0 µM) + ML-7 (0.5 µM) + L-NAME (2 µM) which may afford a similar effect while limiting adverse physiological side effects. On this basis, we proved that intracoronary infusion of the tested mixture protected the heart from IRI, which was reflected by a decreased synthesis of MMP-2 and MMP-9, as well as a significantly reduced extracellular release of cardiac structural and functional proteins, such as MLC1 and BNP-26. Reduced MLC1 and BNP-26 release correlated with increased total antioxidant capacity and glutathione oxidase and reductase activity, pointing to a potential protective pathway.

Analysis of the pro- and antioxidant parameters in the serum revealed a dynamic of change during the peri-infarction period. Data showed a significant increase in the TAC during reperfusion, both in the group subjected only to a MI and those subjected to a MI and MIX treatment. Inversely, prooxidant parameters decreased in the blood during reperfusion (TR), which could be the result of the hyperactivity of antioxidant systems. Some oxidative damaged proteins are not immediately degraded intracellularly but further oxidized, resulting in the intracellular accumulation of lipofuscin. LF, which is a highly oxidized cross-linked aggregate consisting of oxidized protein and lipid clusters [71], in this study, did not reveal significant changes during the time, which excludes it as a useful marker in the peri-infarction period. Comparing the impact of the treatment used, we showed that intracoronary administration of the inhibitory mixture led to decreased TAC during reperfusion, and decreased TOS, OSI and LF before euthanasia in comparison to the MI group. This confirms that intracoronary administration of DOXY, ML-7 and L-NAME significantly reduces oxidative stress during and after reperfusion, which further may reduce reperfusion damage and the area of fibrosis, which undoubtedly translates into cardiac function.

## 5. Conclusions

In conclusion, our study demonstrates that the intracoronary administration of low concentrations of doxycycline in combination with ML-7 and L-NAME is incredibly efficient in increasing the total antioxidative capacity and decreasing the total oxidative status, oxidative stress index, and malondialdehyde concentration. These findings make available a possible application of MIX intracoronary to deal with microvascular dysfunction and heart injury during an MI in the future.

## Figures and Tables

**Table 1:** Analysis of oxidative status in infarcted and non-infarcted heart tissue of the left ventricle. Notes: TOS—total oxidative status; OSI—oxidative stress index; MDA—malondialdehyde; LVIA—left ventricle heart tissue from infarct area; LVCA—left ventricle heart tissue form non-infarct area (control); MI—myocardial infarction; MI-MIX—myocardial infarction with administration of MIX; MIX—mixture of doxycycline (1 μM), ML-7 (0.5 μM) and L-NAME (2 μM), ^a^—LVIA vs. LVCA; data presented as mean ± standard deviation or median (lower- upper quartile).

Oxidative Marker	Heart Tissue Area	MI	*p* Value ^a^	MI-MIX	*p* Value ^a^
TOS [μmol/L]	LVIA	12.2 ± 1.4	<0.001	5.7 ± 0.8	<0.001
	LVCA	6.4 ± 0.9		2.8 ± 1.0	
OSI [index]	LVIA	236.7 (220.7–331.6)	<0.001	75.6 (67.1–82.4)	<0.001
	LVCA	21.7 (19.6–24.5)		5.7 (3.8–6.7)	
MDA [μmol/L]	LVIA	6.4 ± 0.7	<0.001	2.7 ± 0.3	<0.01
	LVCA	1.2 ± 0.2		0.6 ± 0.1	

**Table 2:** Analysis of antioxidative status in infarcted and non-infarcted heart tissue of the left ventricle. Notes: TAC—total antioxidant capacity; act_GPX—activity of glutathione peroxidase; act_GR—activity of glutathione reductase; act_GST—activity of glutathione S-transferase; LVIA—left ventricle heart tissue from infarct area; LVCA—left ventricle heart tissue from non-infarct area (control); MI—myocardial infarction; MI-MIX—myocardial infarction with administration of MIX; MIX—mixture of doxycycline (1 μM), ML-7 (0.5 μM) and L-NAME (2 μM); ^a^—LVIA vs. LVCA; data presented as mean ± standard deviation.

Antioxidant	Heart Tissue Area	MI	*p* Value ^a^	MI-MIX	*p* Value ^a^
TAC [mmol/L]	LVIA	4.8 ± 0.9	<0.001	7.5 ± 0.9	<0.001
	LVCA	29.4 ± 2.2		53.9 ± 2.0	
act_GPX [IU/mg protein]	LVIA	96.5 ± 1.7	<0.001	62.7 ± 3.2	<0.001
	LVCA	40.1 ± 3.0		20.3 ± 2.7	
act_GR [IU/mg protein]	LVIA	5.6 ± 0.3	<0.001	2.7 ± 0.3	<0.001
	LVCA	3.3 ± 0.7		1.8 ± 0.1	
act_GST [IU/mg protein]	LVIA	18.9 ± 1.0	<0.001	18.7 ± 0.4	<0.001
	LVCA	14.7 ± 0.4		12.5 ± 0.3	

**Table 3:** Analysis of oxidative and antioxidative status in heart tissue subjected to MIX. Notes: TAC—total antioxidant capacity; TOS—total oxidative status; OSI—oxidative stress index; MDA—malondialdehyde; act_GPX—activity of glutathione peroxidase; act_GR—activity of glutathione reductase; act_GST—activity of glutathione S-transferase; MI—myocardial infarction; MI-MIX—myocardial infarction with administration of MIX; MIX—mixture of doxycycline (1 μM), ML-7 (0.5 μM), and L-NAME (2 μM).

Parameter	MI	MI-MIX	*p* Value	MI	MI-MIX	*p* Value
	Infarcted Area			Non-Infarcted Area		
TAC [mmol/L]	4.8 ± 0.9	7.5 ± 0.9	<0.001	29.4 ± 2.2	53.9 ± 2.0	<0.01
TOS [μmol/L]	12.2 ± 1.4	5.7 ± 0.8	<0.001	6.4 ± 0.9	2.8 ± 1.0	<0.001
OSI [index]	236.7	75.6	<0.001	21.7	5.7	<0.001
	(220.7–331.6)	(67.1–82.4)		(19.6–24.5)	(3.8–6.7)	
MDA [μmol/L]	6.4 ± 0.7	2.7 ± 0.3	<0.001	1.2 ± 0.2	0.6 ± 0.1	<0.001
act_GPX [IU/mg protein]	96.5 ± 1.7	62.7 ± 3.2	<0.001	40.1 ± 3.0	20.3 ± 2.7	<0.001
act_GR [IU/mg protein]	5.6 ± 0.3	2.7 ± 0.3	<0.001	3.3 ± 0.7	1.8 ± 0.1	<0.001
act_GST [IU/mg protein]	18.9 ± 1.0	18.7 ± 0.4	>0.05	14.7 ± 0.4	12.5 ± 0.3	<0.01

**Table 4:** Contrast analysis of oxidative and antioxidative status in infarcted and non-infarcted heart tissue of the left ventricle. Notes: TAC—total antioxidant capacity; TOS—total oxidative status; OSI—oxidative stress index; MDA—malondialdehyde; act_GPX—activity of glutathione peroxidase; act_GR—activity of glutathione reductase; act_GST—activity of glutathione S-transferase; LVIA—left ventricle heart tissue from infarct area; LVCA—left ventricle heart tissue from non-infarct area (control); MI—myocardial infarction; MI-MIX—myocardial infarction with administration of MIX; MIX—mixture of doxycycline (1 μM), ML-7 (0.5 μM), and L-NAME (2 μM).

Oxidative/Antioxidative Marker	Heart Tissue Area	*p* Value		
		MI vs. MI-MIX	MI	MI-MIX
TAC [mmol/L]	LVIA	<0.01	<0.001	<0.001
	LVCA	<0.001		
TOS [μmol/L]	LVIA	<0.001	<0.001	<0.001
	LVCA	<0.001		
OSI index	LVIA	<0.001	<0.001	<0.001
	LVCA	<0.001		
MDA [μmol/L]	LVIA	<0.001	<0.001	<0.001
	LVCA	<0.01		
act_GPX [IU/mg protein]	LVIA	<0.001	<0.001	<0.001
	LVCA	<0.001		
act_GR [IU/mg protein]	LVIA	<0.001	<0.001	<0.001
	LVCA	<0.001		
act_GST [IU/mg protein]	LVIA	0.722	<0.001	<0.001
	LVCA	<0.001		

## Data Availability

Data presented in the study are available upon request from the corresponding author.
